# Preventive effect of *Coriandrum sativum* on neuronal damages in pentylentetrazole-induced seizure in rats

**Published:** 2017

**Authors:** Mojtaba Pourzaki, Mansour Homayoun, Saeed Sadeghi, Masoumeh Seghatoleslam, Mahmoud Hosseini, Alireza Ebrahimzadeh Bideskan

**Affiliations:** 1 *Department of Anatomy and Cell Biology, Faculty of Medicine, Mashhad University of Medical Sciences, Mashhad, Iran *; 2 *Pharmacological Research Center of Medicinal Plants, School of Medicine, Mashhad University of Medical Sciences, Mashhad, Iran*; 3 *Neurocognitive Research Center, School of Medicine, Mashhad University of Medical Sciences, Mashhad, Iran*; 4 *Microanatomy Research Center, Mashhad University of Medical Sciences, Mashhad, Iran*

**Keywords:** Coriandrum sativum, Seizure, Apoptosis, Neuronal, Damage, Hippocampus

## Abstract

**Objective:**

*Coriandrum sativum* (*C. sativum*) as a medicinal plant has been pointed to have analgesic, hypnotic and anti-oxidant effects. In the current study, a possible preventive effect of the hydro-alcoholic extract of the plant on neuronal damages was examined in pentylenetetrazole (PTZ) rat model of seizure.

**Materials and Methods:**

Forty male rats were divided into five main groups and treated by (1) saline, (2) PTZ: 100 mg/kg PTZ (i.p) and (3-5) 50, 100 and 200 mg/kg of hydro-alcoholic extract of *C. sativum *during seven consecutive days before PTZ injection. After electrocorticography (ECoG), the brains were removed to use for histological examination.

**Results:**

All doses of the extract reduced duration, frequency and amplitude of the burst discharges while prolonged the latency of the seizure attacks (p<0.05, p<0.01, and p<0.001). Administration of all 3 doses of the extract significantly prevented from production of dark neurons (p<0.01, and p<0.001) and apoptotic cells (p<0.05, p<0.01, and p<0.001) in different areas of the hippocampus compared to PTZ group.

**Conclusion:**

The results of this study allow us to conclude that *C. sativum*, because of its antioxidant properties, prevents from neuronal damages in PTZ rat model of seizure.

## Introduction

A seizure attack is generally characterized by behavioral changes and physical features which are followed by an episode of abnormal brain electrical activity and although it is a main feature of epilepsy disease, it also occurs in other conditions including hypoglycemia, hypocalcaemia and fever (Fisher et al., 2014 ▶; Fisher et al., 2005 ▶). Nowadays, epileptic seizures are fully considered for their disabling complications including brain damage (Holmes, 2002 ▶; Leeman-Markowski and Schachter, 2016 ▶; Masur et al., 2013 ▶). The cell loss and neural damages which occur after seizure attacks are secondary to excessive excitability, massive depolarization of the neurons, excessive calcium entry into the nerve cells, and excessive glutamate release (Stefan and Steinhoff, 2007 ▶). In an excessive release of glutamate, dark neurons are produced in the cerebral and hippocampal tissues; however, these effects are reversible and in certain circumstances can be repaired (Holopainen, 2008 ▶; Kherani and Auer, 2008 ▶; Mikati et al., 2003 ▶). It has also been evidenced that recurrent and even a single convulsive seizure induced by a gamma-amino butyric acid (GABA) inhibitor such as pentylenetetrazole (PTZ) lead to neuronal damage and cell death in form of appearance of dark neurons in different areas of the brain including pyramidal layer of cortex, reticular formation, hippocampus and limbic system (Ebrahimzadeh Bideskan et al., 2015 ▶; Homayoun et al., 2015 ▶; Mansouri et al., 2013 ▶; Seghatoleslam et al., 2015 ▶). Occurrence of dark neurons in the pyramidal layer of cortex, reticular formation of pontine, amygdala, hippocampus and other structures of the limbic system has been pointed in other animal models including seizures like pilocarpine and 4-aminopyridine (4-AP) induced seizures (Baracskay et al. 2008 ▶; Poirier et al., 2000 ▶). Besides epilepsy and seizures, the dark neurons which are hyperargyrophil and hyperbasophil cells with hyperelectron density properties, occur in other conditions such as ischemia, hypoglycemia and head injuries (Zsombok et al., 2005 ▶). Moreover, neuronal apoptosis in the hippocampus following seizures induced by epileptogenic substances including pilocarpine and PTZ has been well documented (Israels and Israels, 2000 ▶; Meldrum, 2002 ▶; Naseer et al., 2011 ▶; Naseer et al., 2009 ▶; Pohle et al., 1997 ▶). Epilepsy and seizures are accompanied by production of free radicals in most cases which leads to lipid peroxidation, brain tissues oxidative damage and finally neuronal damage (Choopankareh et al., 2015 ▶; Gridling et al., 2010 ▶; Vafaee et al., 2015 ▶). Neuroprotective effects of natural products with anti-oxidant properties have been fully documented in epilepsy and seizure (Homayoun et al., 2015 ▶; Hosseini et al., 2013 ▶; Naseer et al., 2011 ▶; Tomé et al., 2010; Vafaee et al., 2015 ▶). Additionally, some of medicinal plant extracts have beneficial effects on seizure which are attributed to their anti-oxidant effects (Homayoun et al., 2015 ▶; Hosseini et al., 2013 ▶; Vafaee et al., 2015 ▶). Among medicinal plants, the anti-oxidant effects of *Coriandrum sativum* (*C. sativum*), (coriander) especially in the brain are considerable (Deepa and Anuradha 2011 ▶; Karami et al. 2015 ▶). These benificail effects are suggested to be superior to the well-known antioxidant agents like ascorbic acid (Harsha and Anilakumar, 2014 ▶). This plant, also known as Chinese parsley, and cilantro is an annually grown herb that belongs to the Apiaceae family. It is mostly found in Mediterranean countries and currently is cultivated in many other countries (Laribi et al. 2015 ▶). The plant is freshly used as a vegetable (Karami et al., 2015 ▶); also, fresh leaves and dried seeds of this plant are extensively used in folk medicine (Burdock and Carabin, 2009 ▶). It is traditionally believed that coriander relieves pain and treat anxiety, flatulence and is recommended for loss of appetite (Zargar-Nattaj et al., 2011 ▶). *C. sativum* contain a wide range of compounds including quercetin 3-glucoronide, linalool, camphor, geranyl acetate, geraniol and coumarin (Hosseinzadeh and Madanifard, 2000 ▶). Several studies have confirmed that both the leaves and the seeds of coriander have antimicrobial, anti-inflammatory and anti-cancer effects (Hosseinzadeh and Madanifard, 2000 ▶; Laribi et al., 2015 ▶; Wangensteen et al., 2004 ▶). It was formerly shown that the plant extract has sedative and muscle relaxant effects (Emamghoreishi et al., 2005 ▶; Rakhshandeh et al., 2012 ▶). Anticonvulsant effect of *C. sativum* seeds extracts against PTZ- induced seizures and maximal electroshock in mice has been reported (Hosseinzadeh and Madanifard, 2000 ▶). Moreover, the results of a behavioral study revealed that the hydro-alcoholic extract of aerial parts of the plant have anticonvulsant effects in PTZ model which were accompanied with a protective effect against brain tissues oxidative damage (Karami et al., 2015 ▶). Given the widespread use of coriander with various functions in traditional medicine and increasingly interest of researchers to discover the medicinal effects of various plants, in the present study the effect of hydro-alcoholic extract of coriander was assessed using ECoG criteria (latency, amplitude, duration, and frequency of burst discharges) and neuronal damage of hippocampal formation in PTZ-induced seizure model.

## Materials and Methods

**Preparation of extract**

The leaves, stems, and twigs of *C. sativum* were collected from Neyshabour region, Khorasan Razavi Province, Iran and authenticated by botanists of School of Pharmacy, Mashhad University of Medical Sciences, Mashhad, Iran. A voucher specimen (Herbarium No: 10068) for further reference was deposited at the herbarium center of the same department. To prepare the hydro-alcoholic extract, the plant materials (50 g) were dried and extracted with 300 ml ethanol-water (70/30, v/v) using a Soxhlet apparatus. The resulting extract was concentrated under reduced pressure with a rotatory vacuum evaporator (Karami et al., 2015 ▶) and kept at -4˚C until being used.

**ECoG recordings and PTZ injection**

The animals were anesthetized with ketamine hydrochloride 100 mg/kg and xylazine 20 mg/kg (Dong et al., 2013 ▶; Homayoun et al., 2015 ▶) and placed in a stereotaxic frame. After making holes in the skull, two silver electrodes were implanted on the dura mater of the left and right somatosensory cortex (Karimzadeh et al., 2012 ▶) and a reference electrode was put on the nasal bone. ECoG was recorded using a custom-made deferential ampliﬁer (with band-pass ﬁlters at 0.5–30 kHz, sampling rate 10 kHz, and 0.3–100 Hz (EXT-02 F, NPI, Germany)) and stored by a digital oscilloscope. Recordings were performed for 10 min before and 30 min after a single injection of PTZ (100 mg/kg dissolved in saline, i.p.) (Sigma, USA) (Homayoun et al., 2015 ▶; Karimi et al., 2015 ▶; Karimzadeh et al., 2012 ▶). Latency, amplitude, duration, and frequency of spikes were calculated using AxoScope software.

**Animals and the experimental protocol**

Forty adult male Wistar rats (weighing 200-250 g), were housed under a temperature (22±1 °C ) and an illumination times of 7:00 a.m. to 7:00 p.m., with food pellets and water available *ad libitum*. Animal handling and all experimental protocols were carried out according to Mashhad University of Medical Sciences Ethical Committee Acts. Animals were arbitrary divided into five groups as the following: Control group received saline (one week) instead of coriander extract or PTZ but underwent the surgery procedure and electrode implantation without ECoG recording (n= 8). The animals in PTZ group were treated with saline instead of the extract for one week and were then injected with a single dose of PTZ (100 mg/kg, i.p.) and underwent the surgery procedure and recording (n= 8). The animals of other experimental groups including Ext 50-PTZ, Ext 100-PTZ and Ext 200-PTZ (n=8 in each group) were treated with 50, 100, and 200 mg/kg (i.p.) of the extract, respectively, for one week before PTZ injection and ECoG recordings (latency, amplitude, duration, and frequency of burst discharges).

**Histological assessment**

Two hours after completion of ECoG, all rats in different groups were injected with an overdose of ketamine and then, transcardially perfused with 100 ml of cold PBS followed by 100 ml of cold fixative solution (10% formalin in 0.1 M phosphate buffer, pH = 7.4). Afterwards, the brains were removed and kept in 10% formalin for 48 hr. Then, dehydration, clearing, and paraffin embedding were done, respectively. Coronal sections with thickness of 8 μm were cut every 100 micron from 2.3 to 4.3 mm posterior to the bregma (the boundary of the hippocampus according to the atlas of Paxinos and Watson) (Sadeghian et al., 2012 ▶). An average of 10 equally distant slices of every block from brain of each animals was selected for TUNEL assay (Ebrahimzadeh et al., 2011 ▶). Then, for dark neuron detection, sections were stained with toluidine blue. All slides were observed with light microscope (BX51, Japan) using 40x objective lens (UPlan FI, Japan) and images were captured digitally from different regions of the hippocampus including CA1, CA2, CA3, and DG (dentate gyrus) of both hemispheres.

**Apoptotic cell detection**

To detect apoptotic cells, DNA fragmentation of nuclei was determined in apoptotic cell using TUNEL assay (Terminal deoxynucleotidyl transferase mediated dUTP Nick End Labeling). In the beginning, sections were deparaffinized, cleared with xylene, and rehydrated through descending concentrations of ethyl alcohol and rinsed in 0.1 M PBS for 10 min. After washing in 0.1 M PBS, the sections were treated with 20 µg/ml proteinase K for 20 min at ambient temperature. In order to endogenous peroxidase activity blockage, the specimens were dipped in hydrogen peroxide (3% in methanol) solution in dark for 10 min and then rinsed in 0.1 M PBS (3 times for 5 min) at room temperature. Thereafter, slides were incubated in the TUNEL reaction mixture (Label solution and Enzyme solution) at 4°C, overnight. After incubation, all the slides were washed in PBS and then horseradish peroxidase (POD, 1:500) for 1 hr at room temperature was applied. After extensive washing in PBS (3 min), in order to staining the cells, diaminobenzidine (DAB) was applied for 15 min at ambient temperature in dark. Finally, slides were stained with Harris hematoxylin and cover slipped. Positive and negative controls were as follow: positive control sections were incubated with DNase I (3000 U/ml in 50 mM TrisHCl, pH 7.5, 1 mg/ml BSA) for 10 min at 15-25°C to induce DNA strand breaks, then, TUNEL reaction was applied. Negative control sections were incubated with just Label solution (without terminal transferase) instead of TUNEL reaction mixture (Ataei and Ebrahimzadeh, 2014 ▶).

**Quantiﬁcation analysis**

For quantitative analysis of dark neurons and TUNEL positive cells per unit area (N_A_) of the CA1, CA2, CA3 and DG subdivisions of the hippocampus, the morphometerical method was used (Seghatoleslam et al., 2013 ▶). All selected sections were digitally photographed and the number of dark neurons and TUNEL positive cells were computed by a 10000 μm^2^ counting frame. The mean number of neurons (N_A_) in different areas of the hippocampus was calculated using the following formula:

$N_{A}=\frac{\sum \overset{\text{̅}}{Q}}{\frac{a}{f}.\sum P}$

In mentioned formula, $"\sum \overset{\text{̅}}{Q}"$ is the summation of counted neurons appeared in sections, "a/f" is the area associated with each frame (10000 μm^2^), $"\sum \overset{\text{̅}}{Q}"$ is the summation of frames associated points hitting the reference (Howard and Reed, 2004 ▶). 

**Statistical analysis**

All data were given as mean ± SEM. Analysis of variance (ANOVA) followed by Tukey’s *post hoc* test was carried out for comparing of the data of different groups. The statistical signiﬁcance was attained when p< 0.05. 

## Results

**The effect of coriander extract on PTZ-induced seizures**

**Figure 1:**
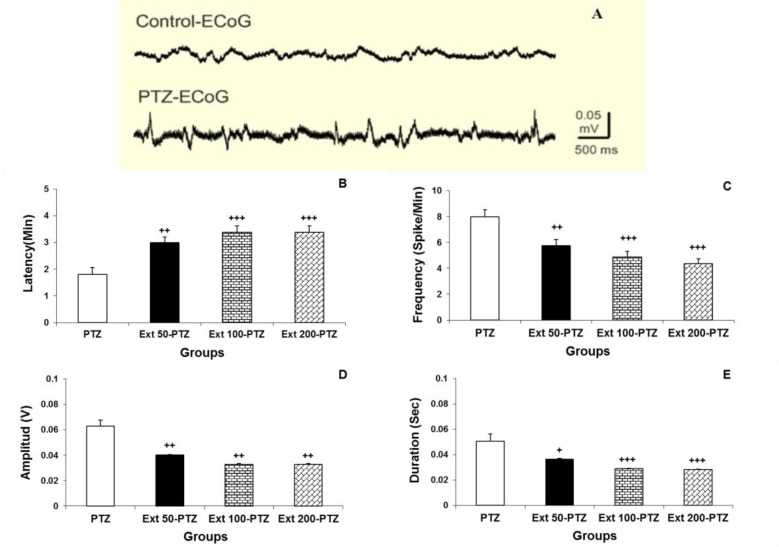
Comparing epileptiform burst discharges in PTZ and control groups which were recorded by the electrocorticogram (A). The latency in all pretreatment groups was significantly more than PTZ group (B). The frequency of burst discharges in pretreatment groups was significantly less than PTZ group (C). The amplitude of burst discharges in Ext 50-PTZ, Ext 100-PTZ and Ext 200-PTZ groups was also significantly lower than PTZ group (D). Finally, the duration significantly increased in pretreatment groups as compared to PTZ group (E). ^+ ^p<0.05, ^++^ p<0.01 and ^+++^ p <0.001 compared to PTZ group

Seizure induced by PTZ (100 mg/kg) in anesthetized rats typically started with vibrissae twitching and facial myoclonus, followed by generalized tonic-clonic convulsions of four limbs. ECoG was monitored to confirm the seizure occurrence. Epileptiform burst discharges were perceived during seizure attacks (Figure 1A). The latency of seizure attack, frequency, amplitude and duration of these burst discharges in PTZ group were 1.8 ± 0.25 min, 8 ± 0.55/ min, 0.063 ± 0.005 V and 0.05 ± 0.005 sec, respectively. Administration of coriander extract at the doses of 50, 100 and 200 mg/kg significantly prolonged the appearance of seizures to 3 ± 0.20 min, 3.4 ± 0.24 min, and 3.4 ± 0.14 min, respectively (p<0.001; Figure 1B). Moreover, different doses of coriander extract significantly decreased the amplitude, duration, and frequency of epileptiform burst discharges induced by PTZ injection (p<0.05). The frequency of the seizure discharges in all three groups of the animals treated with coriander was declined to 5.75 ± 0.48, 4.88 ± .043, and 4.37 ±0.38 per minute, respectively (p<0.001; Figure 1C). The amplitude of the epileptiform discharges in all three groups of animals treated with all selected doses of coriander extract was reduced to 0.04 ± 0.005, 0.033 ± 0.005, and 0.33 ± 0.004 V, respectively (p<0.01; Figure 1D). The duration of PTZ-induced burst discharges was also considerably fell to 0.036 ± 0.004, 0.029 ± 0.004, and 0.028 ± 0.003 sec after administration of all selected doses of coriander extract, respectively (p<0.01; Figure 1E). Although there was no significant difference between 100 and 200 mg/kg doses of coriander extract on the ECoG criteria, it was shown that these two doses significantly changed the latency, frequency and duration of epileptiform burst discharges as compared to 50 mg/kg coriander extract (p<0.05; Figures1B, C, D, and E).

**The effect of coriander extract on the number of dark neurons**

**Figure 2:**
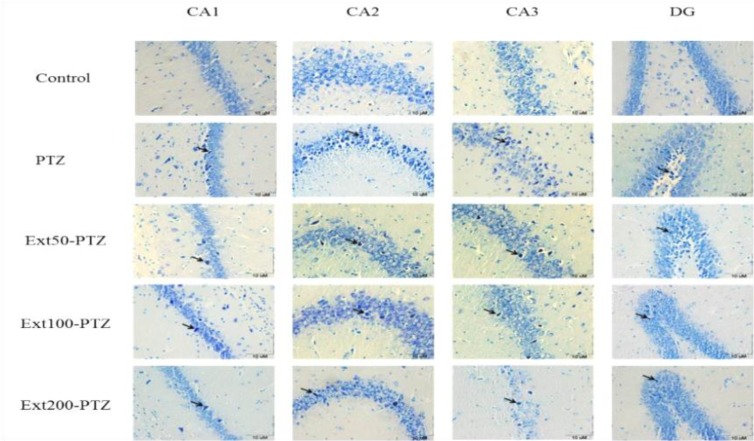
Coriander (50, 100 and 200 mg/kg) restrained dark neurons production in the hippocampal regions in rats after induction of seizure by PTZ injection. Light-microscopic images of normal pyramidal cells and dark neurons that stained by toluidine blue are shown. All photos are in coronal sections of the hippocampal regions in different groups. Arrows show dark neurons among normal pyramidal cells.

**Figure 3:**
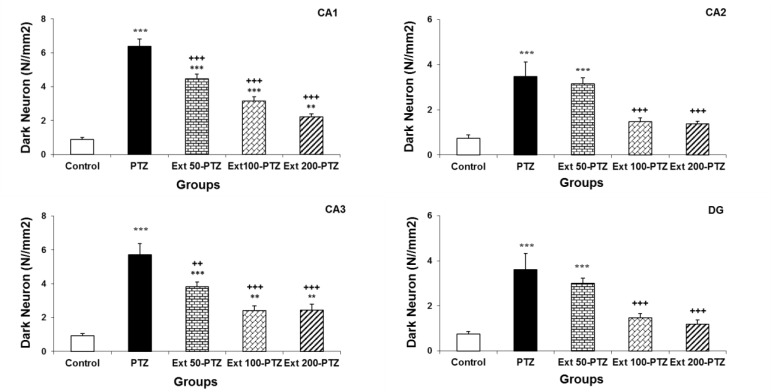
The inhibitory effect of hydro-alcoholic extract of *C. sativum* on dark neurons production. Administration of coriander extract (100, and 200 mg/kg) significantly prevented the production of dark neurons in various areas of hippocampus after induction of seizure by PTZ. Injection of extract at the dose of 50 mg/kg caused significant differences in number of dark neurons only in CA1 and CA3 regions. ^++ ^p<0.01 and ^+++^ p<0.001 compared to PTZ group.^ **^p<0.01 and ^***^p<0.001 compared to control group.

Dark neurons were observed in various subdivisions of the hippocampus in all tested groups, but they were more abundant in the PTZ group (Figure 2). The number of dark neurons per unit area (N_A_) in different regions of the hippocampus (CA1, CA2, CA3 and DG) in control group was 1.8 ± 0.25, 1.5 ± 0.3, 1.8 ± 0.3 and 1.5 ± 0.2, respectively. Injection of PTZ significantly enhanced the mean number of dark neurons in the same areas of the hippocampus to 12.8 ± 0.8, 6.9 ± 1.3, 11.4 ± 1.3 and 7.2 ± 1.4, respectively (p<0.001; Figure 3). The results also showed that the mean number of dark neurons in CA1 and CA3 was considerably more than CA2 and DG (p<0.05). *C. sativum* extract administration at 50-200 mg/kg doses remarkably restrained production of dark neurons by PTZ injection in all regions and declined the mean number of dark neurons per unit area (p<0.001; Figure 3). Although 50 mg/kg of the extract substantially decreased the mean number of dark neurons in CA1 and CA3, to 8.9 ± 0.5 and 7.7 ± 0.5, respectively, it did not change the mean number of these cells in CA2 and DG (p=0.5; Figure 3). By injection of 100 mg/kg of coriander, the number of dark neurons per unit area in CA1, CA2, CA3, and DG, reduced by 50% compared to PTZ group. Besides, administration of 200 mg/kg of the extract showed same reduction in the mean number of dark neurons in the same area (p<0.001; Figure 3).

**The effect of coriander extract on the production of TUNEL positive cells**

**Figure 4:**
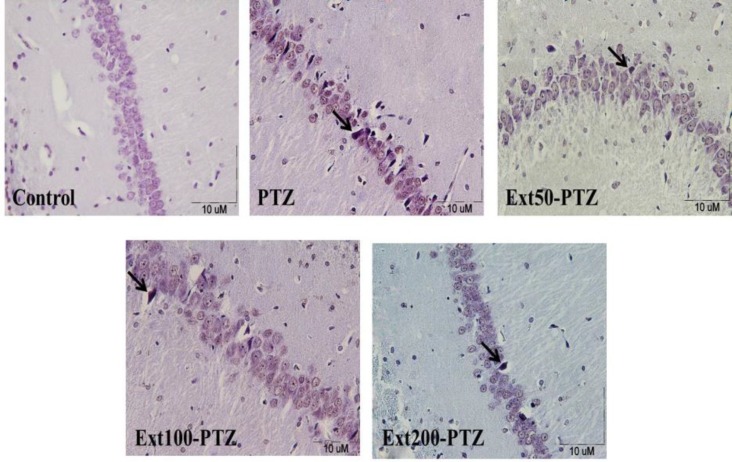
Light-microscopic images of normal pyramidal cells and the TUNEL positive cells (arrows) in coronal sections of rat hippocampus in different groups. TUNEL positive cells which appear in the area have irregular cytoplasms with shrinkage and brown nuclei. A section from control group showing normal pyramidal cells (A). B, C, D and E show the sections from PTZ group and Ext 50-PTZ, Ext 100-PTZ and Ext 200-PTZ groups, respectively.

**Figure 5:**
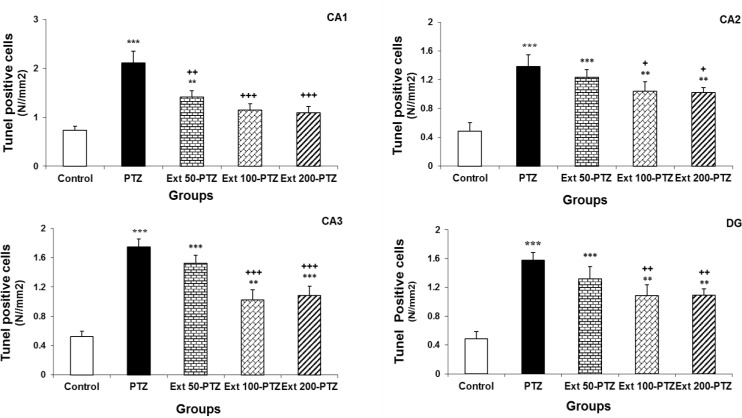
The inhibitory effect of *C.* *sativum* on the apoptotic cells production in regions of the hippocampus (CA1, CA2, CA3, and DG) in rats after induction of seizure by PTZ. TUNEL positive cells in CA1, CA2, CA3, and DG regions of PTZ group were significantly more than control groups. Administration of 50 mg/kg coriander extract significantly declined the mean number of TUNEL positive cells only in CA1 region. By using of 100 and 200 mg/kg of *C.* *sativum,* apoptotic cells number considerably was decreased in all regions. ^+ ^p<0.05, ^++ ^p<0.01 and ^+++ ^p<0.001 as compared to PTZ group. ^**^p<0.01 and ^***^p<0.001 as compared to control group.

Using TUNEL assay, the apoptotic cells that are identified by chromatin condensation, nuclear shrinkage, and DNA fragmentation were detected by the appearance of dark brown nucleus (Figures 4A, 4B, 4C, 4D, and 4E). A few TUNEL positive cells were found in different regions of the hippocampus in control group. By injection of PTZ the number of apoptotic cells per unit area (N_A_) was raised in different regions of the hippocampus in comparison to the control group (p<0.001; Figure 5). Although administration of *C.* *sativum* extract at the dose of 50 mg/kg, noticeably (p<0.01) reduced the mean number of TUNEL positive cells induced by PTZ injection in the CA1 region, it did not change TUNEL positive sells number per unit area in CA2, CA3 and DG reigns (Figure 5; p= 0.1). Injection of 100 mg/kg of coriander extract moderately decreased the mean numbers of TUNEL positive cells in CA1, CA2, CA3, and DG regions (p<0.01; Figure 5). In addition administration of 200 mg/kg of *C.* *sativum* extract markedly reduced TUNEL positive cells number per unit area in CA1, CA2, CA3 and DG regions to 2.18 ± 0.2, 2 ± 0.1, 2.1 ± 0.2 and 2.2 ± 0.2, respectively (p<0.01; Figure 5).

## Discussion

The current study indicates the significant preventive effects of hydro-alcoholic extract of *C. sativum *on hippocampal neurons against seizure as well as its anticonvulsant properties in an animal model. Data showed that pretreatment with coriander significantly enhanced the latency and reduced the frequency, amplitude, and duration of epileptiform burst discharges induced by PTZ injection in rats. Besides, our findings displayed a significant depletion of dark neurons and apoptotic cells in the hippocampus of rats. In traditional medicine, coriander is prescribed for its pain relieving, anti- anxiety, and anticonvulsion activities (Avecina, 1991 ▶). Using PTZ and maximal electroshock models, Hosseinzadeh and Madanifard showed that both ethanolic and aqueous extracts of coriander seeds had a beneficial effect on petitmal and grandmal seizures and prolonged the onset of clonic convulsions (Hosseinzadeh and Madanifard, 2000 ▶). Recently, an anticonvulsant effect of the hydro-alcoholic extract of aerial parts of the plant was also reported in PTZ-induced seizure model in rats (Karami et al., 2015 ▶). In their study, prior to PTZ injections, the experimental groups of rats received 100, 500, and 1000 mg/kg of coriander extract and latent period to the first minimal clonic seizure (MCS), and first generalized tonic-clonic seizure (GTCS) were evaluated. The results showed that the extract enhanced the MCS and GTCS latent periods compared to PTZ group (Karami et al., 2015 ▶). In addition, it was formerly revealed that application of 600 and 800 mg/kg of aqueous and hydro-alcoholic extracts as well as essential oil of coriander significantly increased the latency of clonic and myoclonic convulsions in PTZ-induced seizures in mice (Emamghoreishi and Heidari-Hamedani, 2010 ▶). Consistent with previous studies, lower doses of the plant extract which were used in the present study resulted in a rise in the latency of seizure attacks in animals pretreated with 50, 100, and 200 mg/kg of coriander extract as compared to PTZ group. Furthermore, by ECoG recording our results indicated that these doses of coriander extract declined the duration, amplitude, and frequency of epileptiform burst discharges induced by PTZ injection and confirmed the anticonvulsant effects of the plant extract. The responsible compound(s) for anticonvulsant effects of the plant extract was not evaluated in the present study. Anticonvulsant effects of coriander might be, at least in part, due to activation of coumarin and linalool compounds (Hosseinzadeh and Madanifard, 2000 ▶; Mahendra and Bisht, 2011 ▶) which have marked effects on the central nervous system (CNS). Moreover, *C.* *sativum* has been shown to have flavonoid compounds (Guenther, 1950 ▶). The involvement of GABA neurotransmission in convulsion, sleep, analgesia, and locomotors activity is obvious, and since flavonoids can act on GABAergic system in the brain, (Ramezani et al., 2008 ▶) it might be deduced that these compounds which exist in coriander may have interaction with GABA system and may be involved in the plant's anticonvulsant effect which was seen in the present study. Seizure- induced brain injury is a dynamic process which comprises multiple factors. At the recent time, the role of oxidative stress in the early stage and in the progression of epileptic disorders has been accredited (Costello and Delanty, 2004 ▶). Furthermore, oxidative damage of brain tissue induced by free radicals may contribute to some seizures and epilepsy complications including psychiatric obstacles like anxiety, depression, and memory loss (Reilly et al., 2011 ▶). It has been demonstrated that seizure impairs neuronal structure in hippocampal formations which lead to memory impairment (Kohl et al., 2011 ▶). Former studies also showed that seizures may cause some morphological changes such as production of dark neurons in the brain tissues (Karimzadeh et al., 2012 ▶; Toth et al., 1998 ▶). It has been proposed that brain tissues oxidative damages due to free radicals, glutamate, and aspartate have a role in the production of dark neurons (Kherani and Auer, 2008 ▶). Also it was revealed that glutamate release, excessive excitability, and enhancement of intracellular calcium caused by PTZ- induced seizure, result in cell death (Pavlova et al., 2004 ▶). The results of current study showed that PTZ- induced seizure attacks were followed by production of dark neuron in the hippocampus which were approved by former studies (Homayoun et al., 2015 ▶; Karimzadeh et al., 2012 ▶; Mansouri et al., 2013 ▶). However, different concentrations of coriander extract significantly reduced the number of dark neurons in the hippocampus. 

Furthermore, it was suggested that reactive oxygen species (ROS) which are produced during seizure attacks, induce neuronal cell damages resulting in cell death, apoptosis and necrosis (Shin et al., 2011 ▶). Using both experimental animal and human studies, it has been well documented that seizure attacks lasting for more than 30 minutes are followed by apoptosis and neuronal death (Bengzon et al., 2002 ▶). Seizures increase the intracellular calcium concentration rapidly and considerably which seems to be a powerful trigger for apoptotic chromatin fragmentation (Bengzon et al., 1997 ▶). In addition, it has been evidenced that PTZ-induced seizure causes expression of caspase 3 gene as an active gene involved in programmed cell death (Pavlova et al., 2004 ▶). Although longer periods of seizures consistently produce degenerated neurons, in the present study in line with similar studies, (Bengzon et al., 1997 ▶; Gallyas et al., 2008 ▶) it was indicated that also single seizures lead to apoptotic neuronal death. In addition, our results showed that coriander extract significantly depleted the production of apoptotic cells in different areas of the hippocampus in PTZ-induced seizure rats.

In a recent study, protective effects of coriander on PTZ-induced seizures and the brain tissues oxidative damages were investigated (Karami et al., 2015 ▶). It was shown that the plant extract prevented lipid peroxidation due to seizures in the hippocampal tissues of the rats (Karami et al., 2015 ▶). On the other hand, the brain tissues oxidative damage due to seizure has been well known as an important contributing factor in neural damage and memory loss which could be prevented by antioxidant reagents (Hosseini et al., 2013 ▶; Seghatoleslam et al., 2015 ▶; Tomé et al., 2010). Considering the well-known antioxidant effects of coriander, it seems that the hydro-alcoholic extract of the plant can prevent dark neuron and apoptotic cell production in the hippocampal regions which was pointed in this study. 

In conclusion, the present study indicated neuroprotective, anti-apoptotic and anti-convulsive effects of hydro-alcoholic extract of *C.* *sativum* in PTZ-induced seizure rats. Further studies are required to have a better understanding of the responsible compound(s) and the mechanism(s). 
